# Tunable Thermal Transport in Polysilsesquioxane (PSQ) Hybrid Crystals

**DOI:** 10.1038/srep21452

**Published:** 2016-02-22

**Authors:** Pengfei Li, Sui Yang, Teng Zhang, Ramesh Shrestha, Kedar Hippalgaonkar, Tengfei Luo, Xiang Zhang, Sheng Shen

**Affiliations:** 1 Department of Mechanical Engineering, Carnegie Mellon University, Pittsburgh, 15213, United States; 2 Department of Mechanical Engineering, University of California, Berkeley, 94720, United States; 3 Department of Aerospace and Mechanical Engineering, University of Notre Dame, Notre Dame, 46556, United States; 4 Institute of Materials Research and Engineering, Singapore 117602, Singapore; 5 Department of Physics, King Abdulaziz University, Jeddah 21589, Saudi Arabia

## Abstract

Crystalline polymers have attracted significant interest in recent years due to their enhanced mechanical and thermal properties. As one type of organic-inorganic hybrid polymer crystals, polysilsesquioxane can be synthesized by large-scale and inexpensive so-gel processes with two precursors. In this paper, both octylene-bridged and hexylene-bridged PSQ crystals are characterized with infrared spectroscopy and X-ray crystallography to reveal their super high crystallinity. To study the thermal transport in these unique polymer crystals, we use a suspended micro thermal device to examine their thermal properties from 20 K to 320 K, and demonstrate their tunable thermal conductivity by varying the length of alkyl chains. We also conduct non-equilibrium molecular dynamics simulations to study the phonon behaviors across the hydrogen bond interface. The simulation results demonstrate good agreement with the experimental results regarding both the value and trend of the PSQ thermal conductivity. Furthermore, from the simulation, we find that the anharmonic phonon scattering and interfacial anharmnic coupling effects across the hydrogen bond interface may explain the experimentally observed thermal properties.

Advances in polymer sciences have enabled large-scale synthesis of low-cost polymers with a broad range of properties, leading to various applications ranging from the vulcanized rubber used in the tires of cars to synthetic fibers for creating many common textiles. Bulk polymers are usually in an amorphous form due to the entanglement of polymer molecules, and weak van de Waals bonds between polymer molecules dominate their thermal transport properties. As a result, polymers are generally regarded as thermal insulators with thermal conductivity on the order of 0.1 W/m·K at room temperature^1^. Most attempts for improving polymer thermal conductivity have been focused on fabricating composite materials, in which high thermal conductivity additives, such as metallic nanoparticles^2^,^3^ and carbon nanotubes^4^,^5^, are embedded in polymer matrices. However, the thermal conductivity enhancement of polymer composites is generally limited to within one order of magnitude, significantly below the prediction from the engineering rule of mixing based on effective medium rationale. This discrepancy is attributed to high interfacial thermal resistance between the additives and the polymer matrix.

In contrast with bulk polymers, recent theoretical work suggests that individual polymer molecules, e.g., polyethylene (PE) molecules, are predicted to have extremely high thermal conductivity (e.g., ~350 W/m·K for long PE molecules) because of the strong covalent bonds in molecules^6^,^7^,^8^. To harness the intrinsic high thermal conductivity of individual molecules, the molecules in a polymer need to be aligned like a single crystal. The alignment of polymer molecules can be increased by mechanically stretching bulk samples into fibers or thin films^9^,^10^,^11^,^12^. For example, highly stretched crystalline PE nanofibers were demonstrated to have a high thermal conductivity of ~100 W/m·K^13^. Due to the chain-chain van der Waals interaction induced phonon-phonon scattering within each chain, the thermal conductivity of the PE fibers is lower than the theoretically predicted thermal conductivity (~350 W/mK) of a single PE chain. Nevertheless, it is technically difficult to employ conventional low-cost chemical methods to synthesize polymer crystals with aligned molecules. Hydrogen bond based polymer crystals can be chemically synthesized in a large scale and potentially have a thermal conductivity much higher than that of bulk polymers. In terms of bond strength, hydrogen bonds (bond strength ~5 kcal/mol) have a bond strength that lies between van der Waals bonds (bond strength ~ 1 kcal/mol) and covalent bonds (bond strength on the order of 100 kcal/mol)^14^,^15^,^16^. In this letter, we report the thermal transport measurements and modeling of rationally synthesized hydrogen-bond polysilsesquioxane (PSQ) hybrid crystals, and demonstrate their tunable thermal conductivity by varying the length of alkyl chains.

## Results

**Figure 1:**
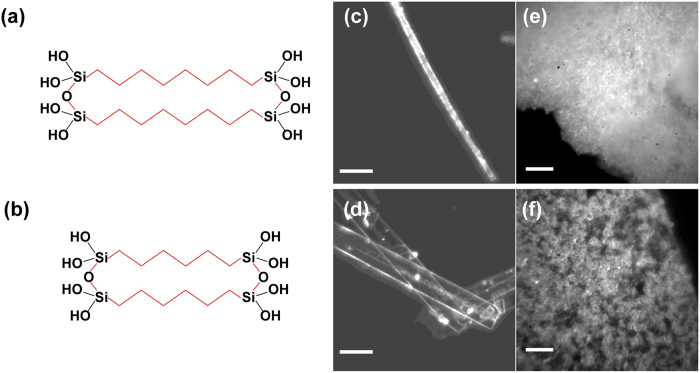
2D projection graphs of repeating units of (**a**) octylene-bridged and (**b**) hexylene-bridged PSQ beams. Dark-field optical images of PSQ products: (**c**) crystalline and (**e**) amorphous octylene-bridged PSQ micro beams; (**d**) crystalline and (**f**) amorphous hexylene -bridged PSQ micro beams. Scale bars in (**c,d**) are 2 μm, and 3 mm in (**e,f**).

As one type of hybrid polymers, the PSQ crystals are organic-inorganic hybrid materials^17^ that have attracted significant interest over the past decades because of their composited and tailored properties from both organic and inorganic components^18^,^19^,^20^. More importantly, it allows sufficient design flexibility to molecular bonding and alignment across intermolecular connections. The PSQ hybrid crystals studied in this work are prepared from two precursors with hexylene- and octylene-bridged groups. As shown in Fig. 1a,b, hexylene-bridged and octylene-bridged crystals are composed of 18- and 22-membered bimolecular rings as repeating units, respectively^21^. The molecular crystalline structures are formed by linking the rings with hydrogen bonds between silanol (Si-O-H) groups and weak van der Waal’s interactions between alkyl chains (See supporting information, Figure S3). In a typical synthesis of crystalline PSQ (Fig. 1c,d), bis-(triethoxysilyl)octane (BTO) or bis-(trimethoxysilyl) hexane (BTH) is dissolved in Tetrahydrofuran (THF) under nitrogen, and the mixture is cooled down to 5 °C. Then, hydrochloric (HCl) acid is added to this solution, allowing hydrolysis of the precursor. The resultant mixture is stirred for 1 hour and kept static for 1 day at the same temperature. Finally, the white product is filtered, washed with ethanol and dried in air. To synthesize amorphous PSQ as a comparison (Fig. 1e,f), BTO or BTH is added to a solution of THF and HCl under nitrogen. The mixture is stirred for 1 hour and kept static for 1 day.

**Figure 2:**
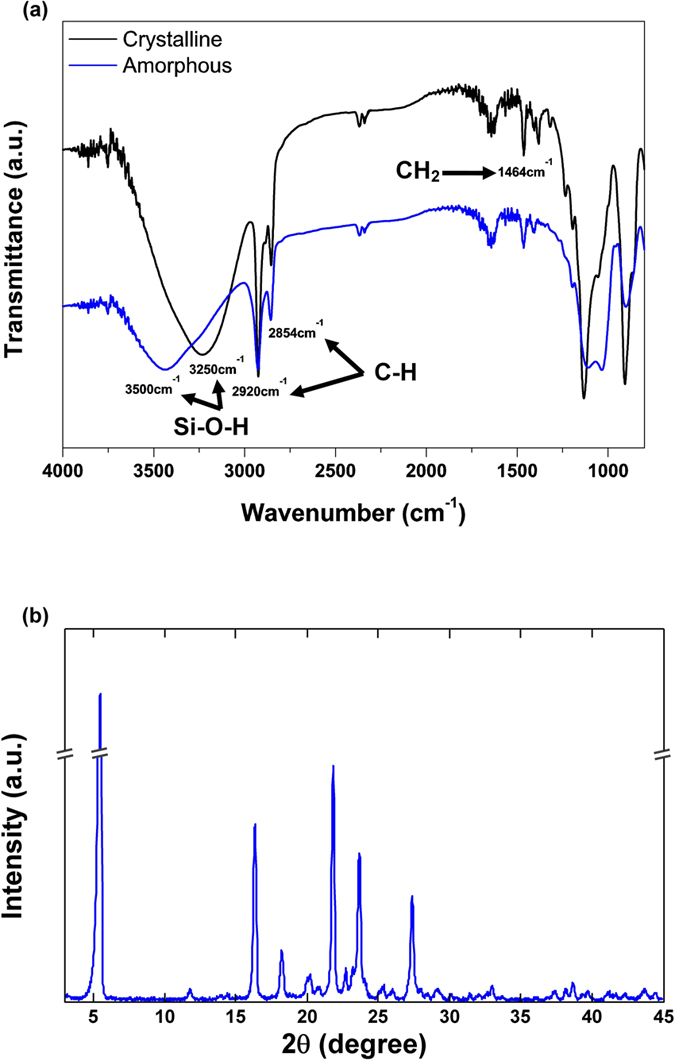
FTIR and X-ray characterizations of PSQ hybrid crystals. (**a**) FTIR spectra of crystalline and amorphous bridged octylene-bridged PSQ. (**b**) Powder X-ray diffraction pattern of octylene-bridged PSQ fibers.

The morphologies of PSQ hybrid crystals are characterized by a standard dark-field optical microscopy with 50x objective (NA = 0.65), as shown in Fig. 1c,d. In comparison with their amorphous counterparts (Fig. 1e,f), octylene-bridged and hexylene-bridged PSQ crystalline beams have more clearly defined geometries and smaller surface roughness. The typical dimensions are 500 nm−2 μm in thickness and 2 μm in width. Figure 2a shows the infrared spectroscopy of octylene-bridged PSQ crystals (similar with that of hexylene-bridged PSQ crystals, Supporting Information), the broadband peak around 3250 cm^−1^ is assigned to the stretching vibration of Si-O-H groups, which indicate that the hydrogen bonded Si-O-H groups restrict the bonding directions^21^. In the amorphous polymer, this broad peak shifts to 3500 cm^−1^ indicating more isolated Si-O-H groups, which render free three-dimensional cross-link amorphous and less dense structure because the degree of condensation is directly related to the number of residual Si-O-H groups^20^. The bands centered at 2920 and 2854 cm^−1^ correspond to the symmetric and anti-symmetric stretching vibration modes, respectively, of the C-H in the methylene unit. The narrow bands in the beam (FWHM = 22 and 13) compared to those in the amorphous structure (FWHM = 43 and 26) indicates the high crystallinity of the internal structure of beams, which is consistent with the observation in the crystalline alkylsiloxanes^22^. The peak at 1464 cm^−1^ corresponds to a CH_2_ scissoring deformation mode, and a singlet at 906 cm^−1^ is assigned to the Si-O-H stretching mode. Several bands observed between 1165 and 1385 cm^−1^ are attributed to coupled CH_2_ wag modes. All these modes in the beam structure are significantly narrower than that of the amorphous structure, which again confirms its high crystallinity. To quantitatively characterize the crystallinity of the PSQ beams, we conduct powder X-ray diffraction (XRD) analysis. Both hexylene-bridged and octylene-bridged PSQ crystals have similarly high crystallinity. For example, the crystallinity of octylene-bridged PSQ crystals is estimated from the XRD pattern (Fig. 2b) to be ~90%.

**Figure 3:**
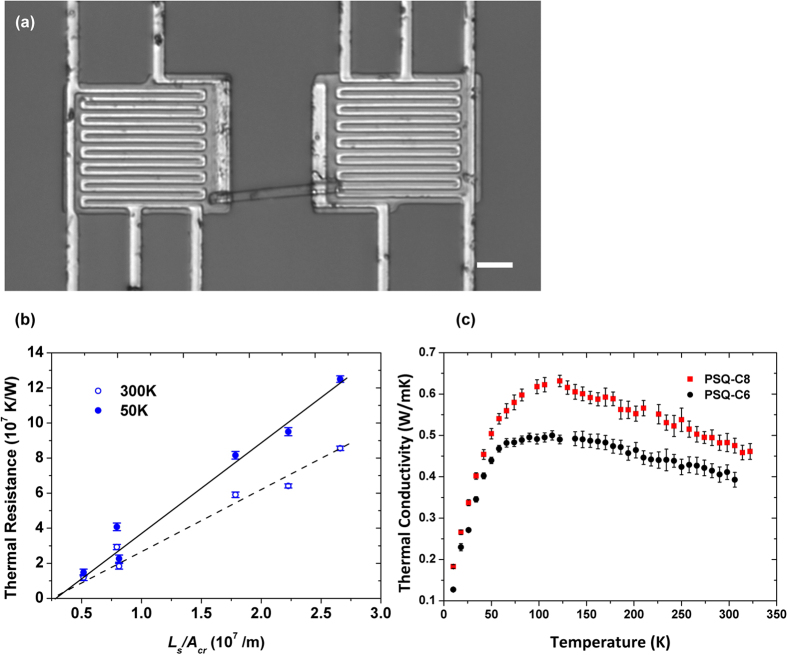
Thermal characterizations of PSQ hybrid crystals. (**a**) Optical image of a PSQ crystal beam placed on a suspended micro device with built-in platinum resistance thermometers. The scale bar is 2 μm. (**b**) The total thermal resistance measured versus *L*_*s*_/*A*_*cr*_ in 5 samples at 50 K and 300 K. (**c**) Thermal conductivity of the two types of PSQ beams at temperatures from 10 K to 320 K.

A variety of methods, such as 3ω method^23^,^24^, Raman spectroscopy^25^,^26^,^27^, and cantilever techniques^13^,^28^, can be used to characterize the thermal properties of materials in micro/nano scale. In our experiment, because PSQ crystals are in regular beam shapes, a suspended micro thermal device is implemented to characterize the thermal properties^29^,^30^,^31^. The device has two suspended SiN_x_ islands fabricated with low pressure chemical vapor deposition and conventional photolithography. On the top of the SiN_x_ islands, a 30 nm thick platinum (Pt) layer is sputtered and patterned to thin serpentine coils. The patterned Pt coil functions respectively as a heater and thermometer for the heating island and a thermometer for the sensing island, while they are isothermal. In Fig. 3a, a PSQ crystal beam is placed on the device using a micromanipulator. In order to reduce the thermal contact resistance, a thermally conductive silver epoxy is applied to fix the two ends of the PSQ beam onto the device. Generally, we use a tungsten tip to bring a small amount of glue onto the metal pads on the heating/sensing islands. Due to good wettability of the expoxy on metals, the expoxy forms a very thin and uniform layer over the metal pads. The PSQ beam is then placed on the heating/sensing islands by another tungsten tip. In order to make sure the PSQ beam does adhere tightly on the pads, after each placement, we attempt to lift the PSQ beam off the pads, and in most cases, the PSQ beam doesn’t come off which demonstrates a good contact between the PSQ beam and the contacting pads.

The micro device together with the sample is placed in a cryostat maintained at a vacuum level below 1 × 10^−7^ Torr. The global temperature *T*_0_ can be precisely controlled via an external temperature controller. At each global temperature *T*_0_, a temperature difference Δ*T* is established between the heating and sensing islands by applying a DC current to the heating island. This temperature difference can be obtained by measuring the electrical resistances and the temperature coefficients of resistance (TCR) of both islands. With the knowledge of the temperature difference Δ*T*, the heat flux *Q*_*s*_ across the PSQ fibers, the fiber length *L*_*s*_ across the two islands and cross section area *A*_*cr*_, the thermal conductivity of the PSQ fibers can be calculated as^32^:


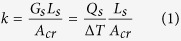


and *G*_*s*_ is the thermal conductance of the fiber.

Consideration of the thermal contact resistance is of great importance in order to determine the PSQ thermal conductivity accurately. The total thermal resistance *R*_*tot*_, the sample thermal resistance *R*_*s,*_ and the thermal contact resistance *R*_*c*_ satisfy the following equation:


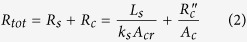


where 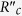 is interface thermal resistance per unit area, *A*_*c*_ is the contact area between the PSQ crystal beam and the Pt pads on both islands. To evaluate the thermal contact resistance, we plot the measured total thermal resistance *R*_*tot*_ versus *L*_*s*_*/A*_*cr*_ of 5 different samples at *T*_0_ = 50 K and 300 K as shown in Fig. 3b. At each temperature *R*_*tot*_ is linear with respect to *L*_*s*_*/A*_*cr*_, and the offsets of the fitted lines are −0.321 × 10^7^ K/W and −0.89 × 10^7^ K/W, which are negligible compared to *R*_*tot*_. Therefore, the thermal contact resistance (*R*_*c*_) between PSQ crystal beams and the Pt Pads can be ignored. Equation (2) can be reduced to be 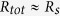 which can then be used to extract the thermal conductivity of the PSQ beam.

Figure 3c shows the typical thermal conductivities of octylene-bridged and hexylene-bridged PSQ beams in the temperature range from 10 K to 320 K. The thermal conductivity of octylene-bridged PSQ beams is generally larger than that of hexylene-bridged PSQ beams, which can be explained by a lower concentration of hydrogen bonds due to a longer octane chain in octylene-bridged PSQ microbeams. The thermal transport in both octylene-bridged and hexylene-bridged PSQ beams clearly exhibits crystal like behaviors. At the temperatures larger than 150 K, the measured thermal conductivities decrease with increasing temperature. This is a signature of the anharmonic Umklapp scattering of phonons in crystals at high temperatures. The temperature dependent thermal conductivity of common high-purity crystals, such as, Si^33^ and GaAs^34^, usually follows the power law *T*^−1^ due to the increase in phonon density. However, for both octylene-bridged and hexylene-bridged PSQ crystals, the thermal conductivity is proportional to *T*^−0.5^ at temperatures larger than 250 K.

To understand the thermal transport in PSQ hybrid crystals, we conduct non-equilibrium molecular dynamics (NEMD) simulations^35^,^36^,^37^,^38^,^39^ to calculate the thermal conductivities of octylene-bridged and hexylene-bridged PSQ beams. The repeating units of both octylene-bridged and hexylene-bridged PSQ beams are first constructed and put in an orthogonal unit cell with periodic boundary conditions in all directions (Table 1). Polymer-Consistent Force Field (PCFF) exported from Material Studio 6 is used to model the molecules^40^. The molecular structures and the unit cells are optimized using multiple algorithms, including Steepest Descent, Conjugate Gradient, and Newton minimization (Smart Minimizer, Discover module of Material Studio 6, Accelrys Inc.). With the minimized structures and unit cells, simulation supercells are obtained by duplicating the unit cell along all three lattice vectors.

**Figure 4:**
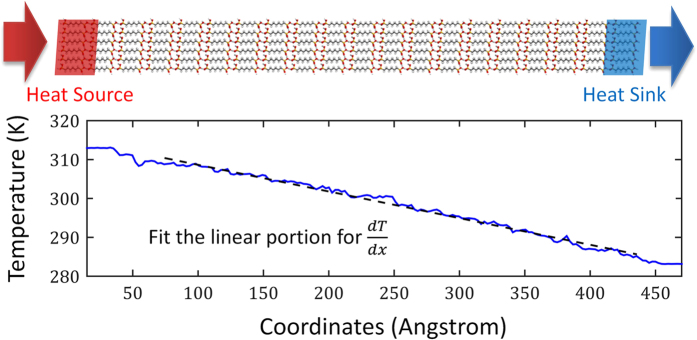
A representative NEMD setup for thermal conductivity calculation.

Figure 4 shows a representative setup and temperature profile of NEMD. In these simulations, a temperature gradient is created and maintained by using Langevin thermostats controlling the temperatures of the two ends of the simulation domain (Fig. 4). The temperatures of the heat sink and heat source regions are set to 15 K lower and higher than the average system temperature, respectively. After steady state is reached, temperature gradient 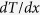 is obtained by fitting the linear portion of the temperature profile, and heat flux can be calculated using 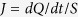, where 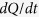 is the average energy change rates of the two Langevin thermostats, and *S* is the cross sectional area. The thermal conductivity is then calculated by Fourier’s law 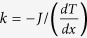. For each simulation, four thermal conductivity values are calculated for different time blocks in the steady state, and the final value is the average of them with the error bar being the standard deviation.

The PSQ beams are first relaxed at 300 K and 1 atmosphere pressure for 1 ns in the constant temperature and constant pressure ensemble. We then fix the volume and run the simulations in constant number of atoms, volume and energy ensemble to calculate the thermal conductivities using NEMD. Thermal conductivity values of two different types of PSQ beams are calculated at 300 K, and they are tabulated in Table 2. It is worth noting that our MD results do not match the exact experimental measured values quantitatively. The cause of quantitative discrepancy can possibly be attributed to the fact that MD simulation modeled a perfect lattice structure while samples in the experiments can contain defects. However, it can be seen that qualitative increasing trend of thermal conductivity from PSQ-C6 to PSQ-C8 is reproduced by the MD simulations and thus our analysis below should shed light on the thermal transport mechanism.

It is well known that thermal transport within one molecule is very efficient due to the well-ordered atomic structure and strong covalent bonds^41^,^42^,^43^. The thermal transport from one molecule to another in the along-beam direction relies on hydrogen bonds between molecules. These interchain hydrogen bonded interfaces are much weaker than the intrachain covalent bonds, and thus the interfaces can work as a bottleneck for thermal transport along the beam. When beams are formed by longer molecules aligning head to tail, the interface density decreases, and thus the effective thermal conductivity of the beam will be higher than that of beam formed by shorter molecules.

**Figure 5:**
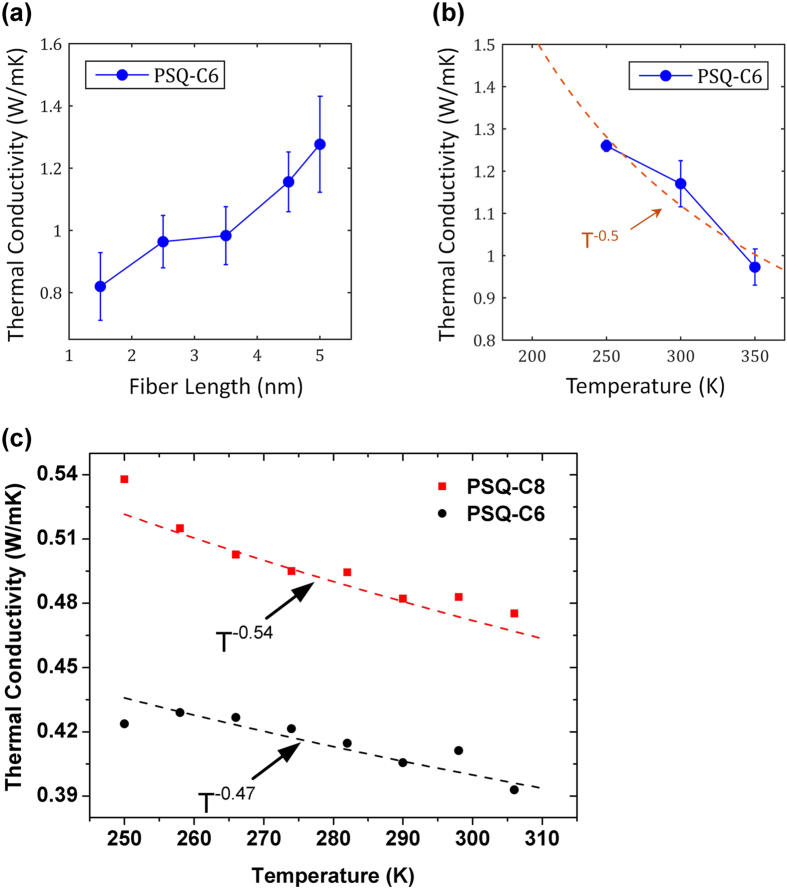
A compare between simulation results and experiment results. (**a**) Simulated thermal conductivity of PSQ-C6 as a function of the beam Length. (**b**) Simulated thermal conductivity of PSQ-C6 from 200 K to 400 K. (**c**) Measured thermal conductivities of PSQ-C6 and PSQ-C8 in the temperature range from 250 K to 310 K.

Due to the periodic arrangement of the molecular chains in the chain length direction, the formation of phonons that has coherent length (i.e., length over which the phase of the heat-carrying phonon is preserved) longer than the length of a molecule may happen. These phonons see the beams as homogenous materials and can travel distances much longer than the length of one molecule. Such a phenomenon is similar to that seen in superlattices^44^,^45^,^46^,^47^,^48^,^49^. To show that such phonons can exist in the PSQ beams due to the periodic structure in the beam, PSQ-C6 beam are simulated with different supercell lengths (Fig. 5a). The increasing trend of the thermal conductivity with respect to the beam length shows that thermal conductivity is influenced by the classical size effect: the mean free paths of phonons are limited by the length of the sample. Such an increasing trend is only possible when the system contains phonons with mean free paths larger than the sample length. This means that not all phonons are scattered diffusively at the hydrogen interfaces but some of them can interfere constructively at the interface and form coherent phonons which travel for much longer distances. It there is no coherent phonons, i.e., all phonons are scattered diffusively at the interfaces between molecules, the thermal conductivity would be a constant disregard of the length of the simulation domain. These phonons usually have long wavelength and mean free path, and will mainly be subject to anharmonic scattering. As the system temperature increases, anharmonic scattering will lower the thermal conductivity (Fig. 5b).

However, there are still some short wavelength phonon modes that are scattered at the hydrogen interfaces. The interfacial thermal transport can be enhanced at higher temperatures due to more anharmonic transport channels being excited^50^. This is especially true for our case in which the interfaces are connected by relative weak hydrogen bonds that should be much more anharmonic than stronger covalent bonds. If we combine the 1/T effect due to anharmonic phonon scattering and the interfacial thermal conductance enhancement effect from temperature, the overall thermal conductivity of the nanobeams should show a dependency weaker than 1/T. Such a combined effect is probably responsible for the experimentally observed *T*^−0.5^ relation at high temperatures (Fig. 5c). MD simulations qualitatively predict the decreasing trend of thermal conductivity and the data from 250 K to 310 K agrees reasonably with the *T*^−0.5^ relation. It is also worth comparing our PSQ fiber with previously reported PE fiber in terms of temperature dependent thermal conductivity. Zhang *et al.*^39^ have previously determined that the thermal conductivity of PE fiber without phase transition indeed displays a *T*^−1^ relation at high temperature. Such a behavior is largely different from the *T*^−0.5^ relation of PSQ fiber thermal conductivity, suggesting that the interface effect is important in the PSQ fibers.

## Conclusion

In summary, a so-gel process with two precursors is adopted to produce the octylene-bridged and hexylene-bridged PSQ crystalline beams. Both the infrared spectroscopy and X-ray diffraction pattern indicate that the hydrogen bond linked PSQ crystal structures have high crystallinity (~90%). We measured the thermal conductivity of hydrogen bond based PSQ beams. The thermal conductivity of the octylene-bridged PSQ beams is measured to be generally higher than the hexylene-bridged ones. The thermal conductivities for both types of beams increase at low temperatures while decreasing at high temperatures, which is attributed to the Umklapp scattering of phonons existing in high-purity crystals. The NEMD simulations reveal that the thermal transport in PSQ crystals displays both coherent phonon transport behavior and interfacial thermal transport feature. The experimental and simulation results in this work demonstrate the tunable and crystal-like thermal transport in PSQ hybrid polymer crystals, which paves the way of practical thermal applications for polymer crystals.

## Additional Information

**How to cite this article**: Li, P. *et al.* Tunable Thermal Transport in Polysilsesquioxane (PSQ) Hybrid Crystals. *Sci. Rep.* **6**, 21452; doi: 10.1038/srep21452 (2016).

## Supplementary Material

Supplementary Information

## Figures and Tables

**Table 1:**
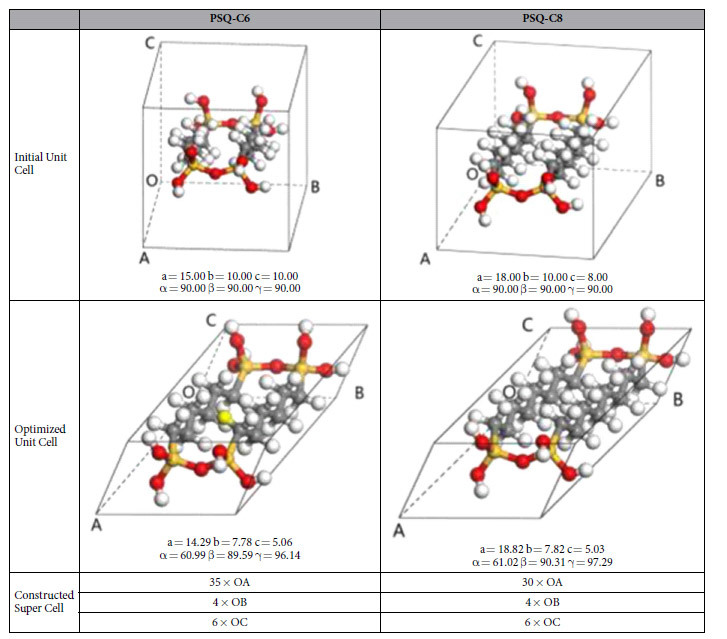
Initial structure and optimized structure for hexylene-bridged (“C6”) and octylene-bridged (“C8”) PSQ crystals.

a = OA, b = OB, c = OC. α = ∠BOC, β = ∠COA, γ = ∠AOB.

**Table 2:** Thermal conductivity of PSQ beams with different molecular backbone lengths at 300 K.

Beam type	Thermal conductivity (W/mK)
PSQ-C8	1.39 ± 0.09
PSQ-C6	1.21 ± 0.03

## References

[b1] Sperling L. Introduction to physical polymer science. (John Wiley & Sons, 2005).

[b2] Jouni M. *et al.* Electrical and thermal properties of polyethylene/silver nanoparticle composites. Polym. Compos. 34, 778–786 (2013).

[b3] Mamunya Y. P., Davydenko V. V., Pissis P. & Lebedev E. V. Electrical and thermal conductivity of polymers filled with metal powders. Eur. Polym. J. 38, 1887–1897 (2002).

[b4] Biercuk M. J. *et al.* Carbon nanotube composites for thermal management. Appl. Phys. Lett. 80, 2767 (2002).

[b5] Marconnet A. M., Yamamoto N., Panzer M. A., Wardle B. L. & Goodson K. E. Thermal conduction in aligned carbon nanotube - Polymer nanocomposites with high packing density. ACS Nano 5, 4818–4825 (2011). 21598962 10.1021/nn200847u

[b6] Henry A. & Chen G. High thermal conductivity of single polyethylene chains using molecular dynamics simulations. Phys. Rev. Lett. 101, 235502 (2008). 19113566 10.1103/PhysRevLett.101.235502

[b7] Hu B., Li B. & Zhao H. Heat conduction in one dimensional chains. Phys. Rev. E 57, 2992–2995 (1997).

[b8] Wang J.-S. & Li B. Intriguing heat conduction of a chain with transverse motions. Phys. Rev. Lett. 92, 074302 (2004). 14995859 10.1103/PhysRevLett.92.074302

[b9] Barham P. J. & Keller A. High-strength polyethylene fibres from solution and gel spinning. J. Mater. Sci. 20, 2281–2302 (1985).

[b10] Smith P. & Lemstra P. J. Ultrahigh-strength polyethylene filaments by solution spinning/drawing, 2(a). Influence of solvent on the drawability. Makromol. Chemie 180, 2983–2986 (1979).

[b11] Greig D. & Sahota M. Thermal conductivity of extruded polyethylene. Polymer. 19, 503–505 (1978).

[b12] Choy C. L., Fei Y. & Xi T. G. Thermal conductivity of gel-spun polyethylene fibers. J. Polym. Sci. Part B 31, 365–370 (1993).

[b13] Shen S., Henry A., Tong J., Zheng R. & Chen G. Polyethylene nanofibres with very high thermal conductivities. Nat. Nanotechnol. 5, 251–5 (2010). 20208547 10.1038/nnano.2010.27

[b14] Zhang L., Chen T., Ban H. & Liu L. Hydrogen bonding-assisted thermal conduction in β-sheet crystals of spider silk protein. Nanoscale 6, 7786–91 (2014). 24811747 10.1039/c4nr01195c

[b15] Schoen P. A. E., Michel B., Curioni A. & Poulikakos D. Hydrogen-bond enhanced thermal energy transport at functionalized, hydrophobic and hydrophilic silica–water interfaces. Chem. Phys. Lett. 476, 271–276 (2009).

[b16] Lodish H. *et al.* Molecular cell biology. (Freeman W. H., 2000).

[b17] Judeinstein P. & Sanchez C. Hybrid organic-inorganic materials: A land of multidisciplinarity chemistry: Synthesis of hybrid materials. J. Mater. Chem. 6, 511–525 (1996).

[b18] Dong F. & Ha C.-S. Multifunctional materials based on polysilsesquioxanes. Macromol. Res. 20, 335–343 (2012).

[b19] Wu L. *et al.* Bithiazole-bridged polysilsesquioxane and its metal complexes: synthesis and magnetic properties. J. Sol-Gel Sci. Technol. 60, 214–220 (2011).

[b20] Shea K. J. & Loy D. A. Bridged polysilsesquioxanes molecular-engineered hybrid organic - inorganic materials. Chem. Mater. 13, 3306–3319 (2001).

[b21] Zhou X. *et al.* Hexylene- and octylene-bridged polysilsesquioxane hybrid crystals self-assembled by dimeric building blocks with ring structures. Chemistry 12, 8484–90 (2006). 16927354 10.1002/chem.200600182

[b22] Parikh A. N. *et al.* n-Alkylsiloxanes: From single monolayers to layered crystals. The formation of crystalline polymers from the hydrolysis of n-Octadecyltrichlorosilane. J. Am. Chem. Soc. 7863, 3135–3143 (1997).

[b23] Cahill D. G. Thermal conductivity measurement from 30 to 750 K: the 3ω method. Rev. Sci. Instrum. 61, 802 (1990).

[b24] Tong T. & Majumdar A. Reexamining the 3-omega technique for thin film thermal characterization. Rev. Sci. Instrum. 77, 104902 (2006).

[b25] Lee J.-U., Yoon D., Kim H., Lee S. W. & Cheong H. Thermal conductivity of suspended pristine graphene measured by Raman spectroscopy. Phys. Rev. B 83, 081419 (2011).

[b26] Yan R. *et al.* Thermal conductivity of monolayer molybdenum disulfide obtained from temperature dependent Raman spectroscopy. ACS Nano 8, 986–993 (2014). 24377295 10.1021/nn405826k

[b27] Stoib B. *et al.* Thermal conductivity of mesoporous films measured by Raman spectroscopy. Appl. Phys. Lett. 104, 161907 (2014).

[b28] Canetta C., Guo S. & Narayanaswamy A. Measuring thermal conductivity of polystyrene nanowires using the dual-cantilever technique. Rev. Sci. Instrum. 85, 104901 (2014). 25362438 10.1063/1.4896330

[b29] Li D. *et al.* Thermal conductivity of individual silicon nanowires. Appl. Phys. Lett. 83, 2934 (2003).

[b30] Lim J., Hippalgaonkar K., Andrews S. C., Majumdar A. & Yang P. Quantifying surface roughness effects onphonon transport in silicon nanowires. Nano Lett. 2475–2482 (2012). 22524211 10.1021/nl3005868

[b31] Hochbaum A. I. *et al.* Enhanced thermoelectric performance of rough silicon nanowires. Nature 451, 163–7 (2008). 18185582 10.1038/nature06381

[b32] Shi L. *et al.* Measuring thermal and thermoelectric properties of one-dimensional nanostructures using a mcrofabricated device. J. Heat Transfer 125, 881 (2003).

[b33] Esfarjani K., Chen G. & Stokes H. T. Heat transport in silicon from first-principles calculations. Phys. Rev. B 84, 085204 (2011).

[b34] Luo T., Garg J., Shiomi J., Esfarjani K. & Chen G. Gallium arsenide thermal conductivity and optical phonon relaxation times from first-principles calculations. Europhys. Lett. 101, 16001 (2013).

[b35] Macgowan D. & Evans D. J. A comparison of NEMD algorithms for thermal conductivity. Phys. Lett. A 117, 5–7 (1986).

[b36] Henry A., Chen G., Plimpton S. J. & Thompson A. 1D-to-3D transition of phonon heat conduction in polyethylene using molecular dynamics simulations. Phys. Rev. B 82, 144308 (2010).

[b37] Luo T., Esfarjani K., Shiomi J., Henry A. & Chen G. Molecular dynamics simulation of thermal energy transport in polydimethylsiloxane. J. Appl. Phys. 109, 074321 (2011).

[b38] Zhang T. & Luo T. High-contrast, reversible thermal conductivity regulation utilizing the phase transition of polyethylene nanofibers. ACS Nano 7, 7592–7600 (2013). 23944835 10.1021/nn401714e

[b39] Zhang T. & Luo T. Morphology-influenced thermal conductivity of polyethylene single chains and crystalline fibers. J. Appl. Phys. 112, 094304 (2012).

[b40] Sun H. COMPASS: An ab Initio force-field optimized for condensed-phase applications s overview with details on alkane and benzene compounds. J. Phys. Chem. B 5647, 7338–7364 (1998).

[b41] Wang Z. *et al.* Ultrafast flash thermal conductance of molecular chains. Science 317, 787–90 (2007). 17690290 10.1126/science.1145220

[b42] Luo T. & Lloyd J. R. Non-equilibrium molecular dynamics study of thermal energy transport in Au–SAM–Au junctions. Int. J. Heat Mass Transf. 53, 1–11 (2010).

[b43] Luo T. & Lloyd J. R. Molecular dynamics study of thermal transport in GaAs-self-assembly monolayer-GaAs junctions with ab initio characterization of thiol-GaAs bonds. J. Appl. Phys. 109, 034301 (2011).

[b44] Luckyanova M. N. *et al.* Coherent phonon heat conduction in superlattices. Science 338, 936–9 (2012). 23161996 10.1126/science.1225549

[b45] Mu X., Zhang T., Go D. B. & Luo T. Coherent and incoherent phonon thermal transport in isotopically modified graphene superlattices. Carbon N. Y. 83, 208–216 (2015).

[b46] Latour B., Volz S. & Chalopin Y. Microscopic description of thermal-phonon coherence: From coherent transport to diffuse interface scattering in superlattices. Phys. Rev. B 90, 1–9 (2014).

[b47] Wang Y., Huang H. & Ruan X. Decomposition of coherent and incoherent phonon conduction in superlattices and random multilayers. Phys. Rev. B 90, 48–50 (2014).

[b48] Ravichandran J. *et al.* Crossover from incoherent to coherent phonon scattering in epitaxial oxide superlattices. Nat. Mater. 13, 168–72 (2014). 24317186 10.1038/nmat3826

[b49] Tian Z., Esfarjani K. & Chen G. Green’s function studies of phonon transport across Si/Ge superlattices. Phys. Rev. B 89, 235307 (2014).

[b50] Wu X. & Luo T. The importance of anharmonicity in thermal transport across solid-solid interfaces. J. Appl. Phys. 115, 014901 (2014).

