# Supplementary material for: Tunable Thermal Transport in Polysilsesquioxane (PSQ) Hybrid Crystals
Source: Sci Rep. 2016 Feb 22;6:21452. doi: 10.1038/srep21452 (PMC4761904; doi:10.1038/srep21452)
Supplement: Supplementary Information [file srep21452-s1.pdf]

Supporting Information

Tunable Thermal Transport in Polysilsesquioxane (PSQ) Hybrid Crystals

Pengfei Li[1], Sui Yang[2], Teng Zhang[3], Ramesh Shrestha[1], Kedar Hippalgaonkar[4,*], Tengfei Luo[3], Xiang Zhang[2], and Sheng Shen[1,*]

[1]Department of Mechanical Engineering, Carnegie Mellon University, Pittsburgh, 15213, United States
[2]Department of Mechanical Engineering, University of California, Berkeley, 94720, United States
[3]Department of Aerospace and Mechanical Engineering, University of Notre Dame, Notre Dame, 46556, United States
[4]Institute of Materials Research and Engineering, Singapore 117602, Singapore
[*]Email: kedarh@imre.a-star.edu.sg
[*]Email: sshen1@cmu.edu

Contents

1. SEM characterization of polysilsesquioxane (PSQ) crystal beams

2. Background heat transfer consideration

3. Comparison between crystalline and amorphous Hexylene-bridged ("C6") samples

4. A 2D projection of a PSQ crystal structure

1. SEM characterization of PSQ crystal beams

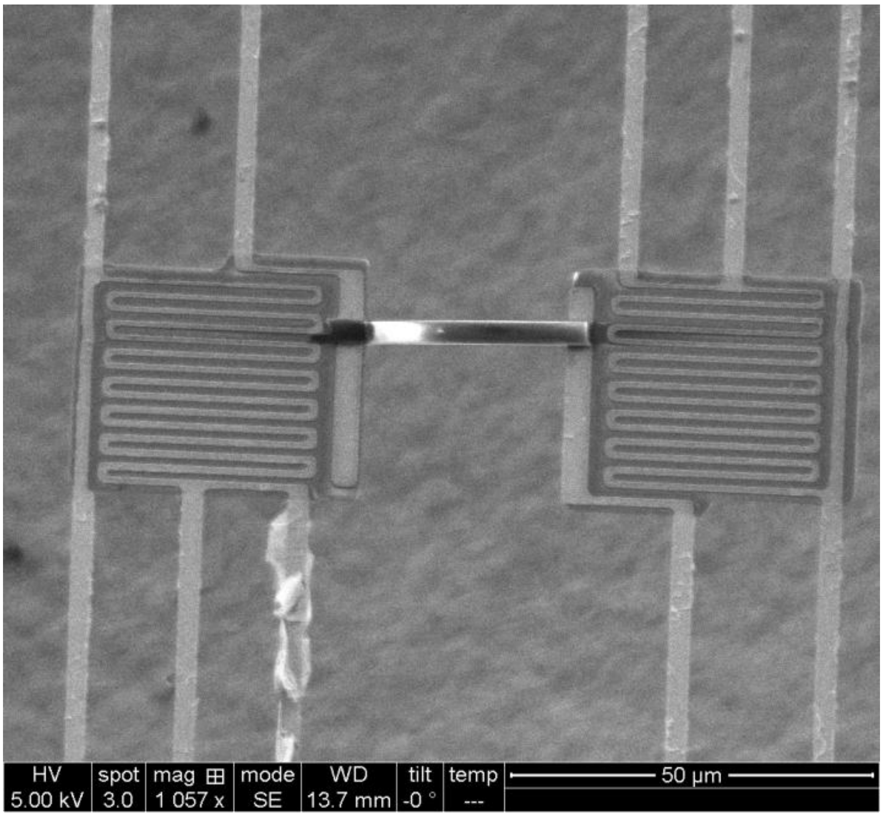

Figure S1. A SEM image of a PSQ beam on the suspended micro device. The brighter region on the PSQ beam is caused by electron accumulation due to intrinsic insulating property of the sample.

2. Background heat transfer consideration

As reported elsewhere [1–3], the background heat transfer between the heating and sensing islands introduces errors and results in an overestimate of the thermal conductance of the PSQ beam. Inspired by previous works, we conduct a control experiment with an empty device

under the same measurement conditions as we measure the PSQ beams. As expected, no noticeable signals are observed from the sensing island. This can be attributed to two reasons: 1) The super high vacuum (below 1×10^{-7} Torr) assures an extremely low concentration of residual gas molecules. 2) The devices used in these measurements have rather large separation gap between the two islands which is at least 15 μm. As we know the view factor of thermal radiation is inversely proportional to the squared distance between the two objects, therefore, the thermal radiation across the two islands is negligible, especially compared with a typical thermal conductance of 7.21×10^{-8} W/K at room temperature. We also consider the heat loss on the PSQ beam due to the thermal radiation to the surrounding. If we ignore the thermal radiation from the surface of the PSQ beam, a relative error below is introduced in the thermal conductivity calculation [1]:

$$\Delta = mL\coth(mL) - 1 \quad (1)$$

where $m = \sqrt{8\epsilon\sigma T_{hs}^3(w+t)/(k_s wt)}$, ϵ, σ,T_{hs} and k_s are emissivity of the PSQ beam, the Stefan-Boltzmann constant, global temperature, and thermal conductivity of the PSQ beam, and w and t are the width and thickness of the PSQ beam. For a typical PSQ beam measure in our experiment, w=2.8 μm, t=1.04 μm, L=16 μm, and let ϵ =0.3, the relative error Δ for ignoring the thermal radiation loss is found to be as small as 0.1%.

3. Comparison between crystalline and amorphous C6 samples

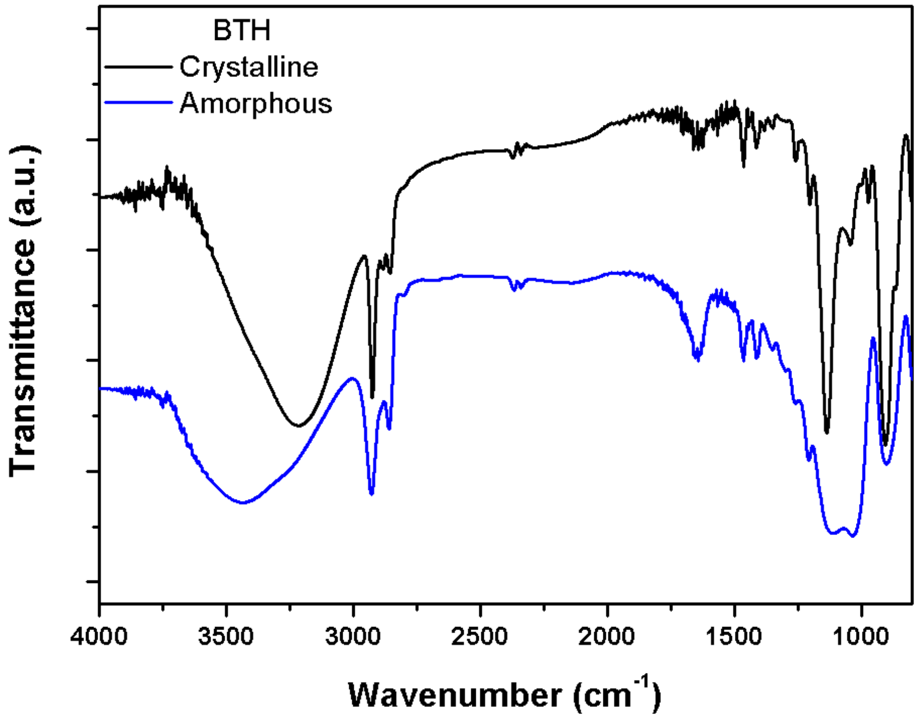

Figure S2. Fourier transform infrared spectroscopy (FTIR) on a C6 PSQ beam prepared with bis-(trimethoxysilyl) hexane (BTH).

4. A 2D projection of a PSQ crstal structure

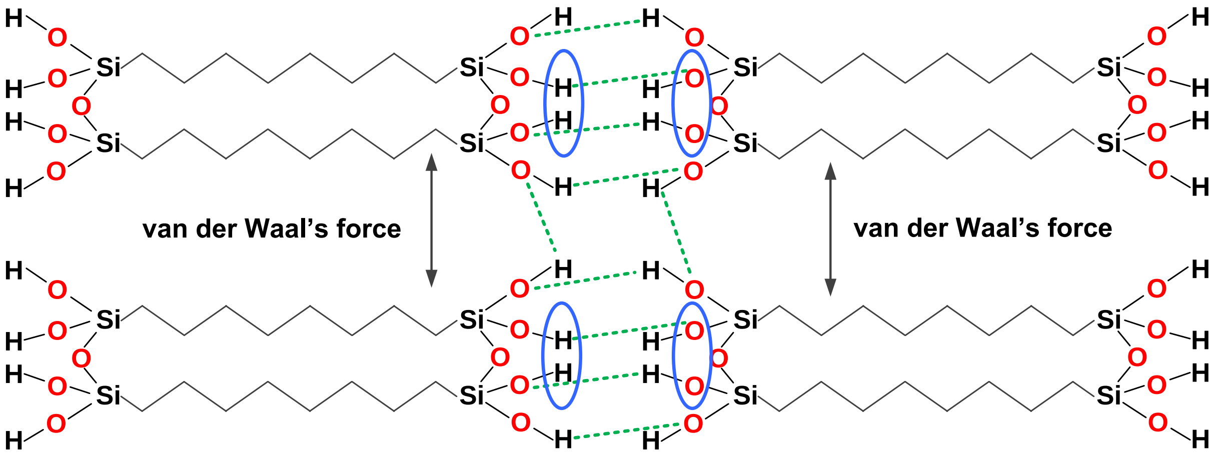

Figure S3. A 2D projection of a PSQ crystal structure formed by the van der Waal's force which binds the sides of the rings and hydrogen bonds which connect the ends of the bimolecular rings. The longitudinal crystal direction is along the carbon chains. The blue circled hydrogen and oxygen are atoms that form hydrogen bonds with molecules in and out paper directions.

References:

1. Weathers, A., Bi, K., Pettes, M. T. & Shi, L. Reexamination of thermal transport measurements of a low-thermal conductance nanowire with a suspended micro-device. *Rev. Sci. Instrum.* **84,** 084903 (2013).

2. Yu, C., Shi, L., Yao, Z., Li, D. & Majumdar, A. Thermal conductance and thermopower of an individual single-wall carbon nanotube. *Nano Lett.* **5,** 1842–1846 (2005).

3. Chen, R. *et al.* Thermal conductance of thin silicon nanowires. *Phys. Rev. Lett.* **101,** 105501 (2008).
